# Validation and generalizability of an asymptomatic bacteriuria metric in critical access hospitals

**DOI:** 10.1017/ice.2024.206

**Published:** 2025-02

**Authors:** Hannah Imlay, Claire E. Ciarkowski, Chloe Bryson-Cahn, Jeannie D. Chan, Whitney P. Hartlage, Adam L. Hersh, John B. Lynch, Natalia Martinez-Paz, Emily S. Spivak, Hannah Hardin, Andrea T. White, Chaorong Wu, Zahra Kassamali Escobar, Valerie M. Vaughn

**Affiliations:** 1 Division of Infectious Diseases, Department of Internal Medicine, University of Utah, Salt Lake City, UT, USA; 2 Veteran’s Affairs Salt Lake City Health Care System, Salt Lake City, UT, USA; 3 Division of General Internal Medicine, Department of Internal Medicine, University of Utah, Salt Lake City, UT, USA; 4 Center for Stewardship in Medicine, University of Washington, Seattle, WA, USA; 5 Division of Allergy and Infectious Diseases, University of Washington School of Medicine, Seattle, WA, USA; 6 Department of Pharmacy, University of Washington, Seattle, WA, USA; 7 Division of Infectious Diseases, Department of Pediatrics, University of Utah, Salt Lake City, UT, USA; 8 Division of Epidemiology, Department of Internal Medicine, University of Utah, Salt Lake City, UT, USA

## Abstract

**Objective:**

Inappropriate diagnosis and treatment of urinary tract infections (UTIs) contribute to antibiotic overuse. The Inappropriate Diagnosis of UTI (ID-UTI) measure uses a standard definition of asymptomatic bacteriuria (ASB) and was validated in large hospitals. Critical access hospitals (CAHs) have different resources which may make ASB stewardship challenging. To address this inequity, we adapted the ID-UTI metric for use in CAHs and assessed the adapted measure’s feasibility, validity, and reliability.

**Design:**

Retrospective observational study

**Participants:**

10 CAHs

**Methods:**

From October 2022 to July 2023, CAHs submitted clinical information for adults admitted or discharged from the emergency department who received antibiotics for a positive urine culture. Feasibility of case submission was assessed as the number of CAHs achieving the goal of 59 cases. Validity (sensitivity/specificity) and reliability of the ID-UTI definition were assessed by dual-physician review of a random sample of submitted cases.

**Results:**

Among 10 CAHs able to participate throughout the study period, only 40% (4/10) submitted >59 cases (goal); an additional 3 submitted >35 cases (secondary goal). Per the ID-UTI metric, 28% (16/58) of cases were ASB. Compared to physician review, the ID-UTI metric had 100% specificity (ie all cases called ASB were ASB on clinical review) but poor sensitivity (48.5%; ie did not identify all ASB cases). Measure reliability was high (93% [54/58] agreement).

**Conclusions:**

Similar to measure performance in non-CAHs, the ID-UTI measure had high reliability and specificity—all cases identified as ASB were considered ASB—but poor sensitivity. Though feasible for a subset of CAHs, barriers remain.

## Introduction

Although guidelines recommend against treatment of asymptomatic bacteriuria (ASB),^1^ clinicians continue to inappropriately prescribe antibiotics.^2–6^ Inappropriate treatment of bacteriuria is associated with worse clinical outcomes, including delays in other diagnoses due to diagnostic anchoring on urinary tract infection (UTI) and increasing antibiotic-associated adverse effects, *C. difficile*, length of stay, selection for drug-resistant organisms, and costs.^7–11^

While antimicrobial stewardship programs (ASPs) have reduced ASB treatment in well-resourced settings, few initiatives have focused on critical access hospitals (CAHs). Centers for Medicare & Medicaid Services (CMS) designate hospitals with fewer than 25 inpatient beds and located >35 miles from another hospital as CAHs. CAHs face significant resource barriers for ASP, including reduced access to infectious diseases specialists, stewardship-trained pharmacists, microbiology resources, and informatics expertise.^12,13^ These resource differences have resulted in the Centers for Disease Control and Prevention issuing CAH-specific implementation guidance highlighting diagnostic considerations for UTIs as a key target for CAH ASPs.^14,15^

Treatment of ASB is common in CAHs,^16^ but currently, there is no standard way to measure, track, or compare their ASB treatment to their peers. One validated national measure, the “Inappropriate Diagnosis of UTI in Hospitalized Patients” measure (ID-UTI), has been used in non-CAHs to improve the percentage of treated ASB relative to all cases of treated bacteriuria.^17–19^ The ID-UTI measure uses a standardized definition of UTI to identify ASB and was found to have high specificity (ie accurate identification of ASB) among cases classified as ASB and high reliability (ie replicability).^20^ The ID-UTI measure has been endorsed by the National Quality Forum for quality improvement in large hospitals, but it has not been assessed in CAHs.

To determine whether the ID-UTI measure could accurately assess ASB vs UTI in CAHs, 10 CAHs within the University of Washington (UW) Center for Stewardship in Medicine (CSiM) consortium^16^ participated in a project to assess the feasibility, validity, and reliability of the ID-UTI measure to quantify treatment of ASB.

## Methods

### Study setting

The UW CSiM pilot was an intensive quality improvement cohort to improve diagnosis and treatment of urinary tract infection, funded by the Office of Rural Health State Flex programs in Arizona, Idaho, Oregon, Utah, and Washington. Of the 19 CAHs who participated in the 1-year pilot, 14 volunteered to participate in a second year both to continue their stewardship work and to help validate the ID-UTI metric in CAHs. Two CAHs dropped out prior to program initiation due to loss of their stewardship champion and two submitted data for 1 month only and were excluded from final analysis. The 10 included CAHs were located in the Pacific or Mountain West region of the United States. Each hospital identified at least one stewardship champion who attended both monthly CSiM education sessions and quarterly one-on-one mentoring sessions to implement a QI project at their hospital. Stewardship champions submitted cases of treated bacteriuria for assessment as UTI vs ASB using the ID-UTI measure definition. Stewardship champions were not physicians and did not have specific training in case submission.

### Inappropriate diagnosis of UTI metric

The ID-UTI measure^20^ quantifies the percentage of treated bacteriuria that is asymptomatic (ASB) relative to all cases of treated bacteriuria. Bacteriuria is defined as a urine culture result flagged “abnormal” by the electronic medical record system at each site. Per the measure, ASB cases are bacteriuric patients who received antibiotics but do not meet the definition for UTI defined as a patient with any of the following signs or symptoms: urgency, rigors, frequency, dysuria, suprapubic pain or tenderness, acute hematuria, costovertebral or flank pain or tenderness, documentation of pyelonephritis, fever >38.0, or new onset mental status changes with systemic signs of infection.

The ID-UTI standard definition of ASB vs UTI was developed based on review of patient cases, an expert panel, and patient focus groups^20^ and has been used to reduce ASB treatment in 69 Michigan Hospitals.^19^ During testing, the measure had high reliability and high specificity though sensitivity was poor (ie the measure under-identified ASB).

### Data sources

From October 1, 2022 through July 31, 2023, participating CSiM CAHs submitted three types of data: a) clinical information for bacteriuria cases treated with antibiotics, b) deidentified patient notes for a randomly selected subset of cases, and c) survey responses regarding metric characteristics.

First, hospitals submitted data on consecutive cases of bacteriuria treated with antibiotics; for some sites, all eligible cases were submitted, for larger sites, a convenience sample of cases was submitted. Because CAHs have fewer than 25 inpatient beds, it was apparent early that focusing on inpatients alone (as was done for the originally validated ID-UTI metric) would not include a large enough sample. Thus, we expanded inclusion criteria to include patients discharged from the emergency department. In the original measure validation process, 59 cases were required to achieve high reliability (0.8), and 35 were required to achieve good reliability (0.7);^17,18,20^ thus, we aimed for 59 case submissions per site over 10 months (or 5–6 cases/month) to achieve high reliability with a secondary goal of 35 cases to achieve acceptable reliability. Consistent with the ID-UTI measure, sites excluded patients who left against medical advice, were admitted to hospice, were pregnant, were <18 years old, had a history of spinal cord injury, received an antibiotic prescription with a duration longer than 14 days (a proxy for identifying a complicated infection), or had a concomitant non-UTI indication for antibiotic therapy. For each eligible case, site champions submitted deidentified data using a REDCap form (see Supplement) that captured data necessary to classify the case as ASB vs UTI using the ID-UTI measure definition, including signs and symptoms of a UTI, vital signs, key demographic characteristics, microbiology, and antibiotic use. An algorithm within the REDCap form identified each case as UTI or ASB based on submitted case characteristics. As a measure of feasibility, we also tracked the amount of time required to input each case into REDCap.

Second, to enable us to assess the validity and reliability of the ID-UTI definition in CAHs, we randomly selected 6 cases (2 cases per quarter) from each hospital for assessment via dual-physician (HI and CC) review. For these cases, each hospital submitted deidentified primary documentation from the medical records including (if present): emergency department note, admission note, discharge note, provider details, vital signs on the day the urine culture was sent, and urine culture results. Based on this information, the two physician reviewers independently assessed each case to determine whether (a) in their clinical opinion, they believed the patient had UTI vs ASB and (b) whether submitted cases met the ID-UTI definition of UTI vs ASB. After independent review, the two clinician reviewers compared their assessments to resolve disagreements. If relevant, the clinician reviewers recorded any reasons their clinical opinion and ID-UTI definition disagreed. Similar to the initial measure validation,^20^ consensus clinical opinion was considered the “gold standard” for validity assessments whereas consensus ID-UTI definition was used for reliability assessments.

Finally, we surveyed CAH champions before and after the data collection period to understand the measure’s face validity (ie the importance and relevance of the ID-UTI measure) and feasibility (ie ease of abstraction). To assess face validity, the pre-intervention survey queried site champions on their beliefs regarding reducing antibiotic treatment for ASB. For feasibility assessment, the pre- and post-intervention surveys queried sites on barriers to data collection. In addition, the post-intervention survey assessed the relative ease of the required data collection (from very easy to very difficult) compared to other quality measures.

### Data analysis

Our primary outcomes were the ID-UTI measure’s validity, feasibility, and reliability in CAHs. (see Table 1 for definitions).

**Table 1.** Definitions of outcomes assessments

Assessment	Definitions^a^	How this was assessed
Feasibility	How well the measure can be captured without undue burden	1. How many hospitals were able to submit the requested number of cases 2. Surveys that assessed barriers to data collection and asked how easy case abstraction was 3. Time taken to input each case into the ID-UTI REDCap tool
Validity	How well the measure correctly reflects the gold standard Face validity: Importance and relevance of the measure	Compared ID-UTI measure to dual-physician review (gold standard) Face validity: Pre-intervention surveys asking about importance of reducing treatment of ASB Outcome metrics: sensitivity, specificity, positive predictive value, negative predictive value
Reliability	How well the measure can be consistently implemented within the organization (i.e., are there errors when cases are input)	Compared abstraction of cases by CAH site representatives with the same process performed by two clinician reviewers Outcome metrics: percent agreement and Cohen’s kappa statistic

a Modified from White et al, 2024^20^

Survey responses and time for case completion are expressed using descriptive statistics and Likert scale categorizations, as appropriate. In addition, feasibility was assessed by quantifying the percentage of CAHs able to submit the total requested number of cases for the following reliability targets: excellent reliability (0.8), 59 cases [goal]; good reliability (0.7, secondary goal), 35 cases; moderate reliability (0.6), 22 cases.^20^

Summary statistics for validity include sensitivity, specificity, percent agreement, positive predictive value (PPV), and negative predictive value (NPV). Confidence intervals were calculated using binomial probabilities.^21^ We also summarized the reasons for differences between clinical opinion and the ID-UTI assessment. Reliability was described by percent agreement and Cohen’s kappa statistics.^22^ R (version 4.4.0) was used for all analyses.

## Results

### Characteristics of participating sites

Of the fourteen CAHs who initially volunteered to participate, four dropped out prior to submitting any data for measure validity/reliability assessment. Two sites dropped out due to lack of a site representative to direct the effort (their prior champion had changed roles) while two sites were unable to collect and submit sufficient cases early in the project. Ten sites participated in the full ten months of the project. Demographics of participating hospitals are shown in Table 2. A total of 608 cases of treated ASB were submitted for ID-UTI assessment with 58 (9%) randomly selected for physician review. Among reviewed cases, the ID-UTI measure classified 16/58 (28%) as ASB.

**Table 2.** Characteristics of participating sites

	Hospital characteristics n = 10 (%)
Location of hospital	
Arizona	1 (10)
Idaho	3 (30)
Oregon	3 (30)
Utah	1 (10)
Washington	2 (20)
Duration of participation in CSiM at the time of project initiation	
>3 yr	2 (20)
2-3 yr	4 (40)
1-2 yr	3 (30)
<1 yr	1 (10)
Availability of infectious disease physician expertise	
None	2 (20)
Available through help line	7 (70)
Available through tele-consultation	1 (10)
Available on-site	0
Professional role of steward champion at each critical access hospital site	
Pharmacist	6 (60)
Nurse	2 (20)
Other^a^	2 (20)
Presence of electronic medical record system (EMR)^b^	10 (100)

a Other roles included compliance director and infection prevention/quality director

b EMRs included Athenahealth alone (n = 1), Meditech alone (n = 1), both Athenahealth and Meditech (n = 1), Epic (n = 2), Cerner (n = 2), Allscripts (n = 1), Centriq (n = 1; transitioned to Epic during the project), and Healthland (n = 1)

### Feasibility

Prior to the intervention, 33% (3/9) of respondents identified time as the major barrier to performing case abstraction and using the NQF metric to guide stewardship interventions, 44% (4/9) identified physician buy-in as a barrier, and 11% (1/9) identified that using the electronic medical record (EMR) to abstract cases was a barrier.

Among the 10 participating sites, 40% (4/10 sites) achieved the goal of >59 cases over 10 months. Another 3 sites achieved our secondary goal of submitting >35 cases (which would achieve “acceptable” reliability of ID-UTI measurement). All 10 sites submitted >22 cases (“moderate” reliability). One site received assistance (H.H.) to successfully abstract cases. The median abstraction time per case was 11 minutes (IQR 7.0 to 18.0).

On post-intervention survey, hospitals identified similar barriers as the pre-intervention survey: time, 43% [3/7]; physician buy-in, 43% [3/7]; and challenges using the EMR, 14% [1/7]). Two of 7 (29%) sites reported the required data collection was “easy” compared to other quality measures and 43% (3/7) of additional sites considered it “neither easy nor difficult.” Additional barriers identified during discussion with sites were: difficulty abstracting cases with existing personnel (n = 1), the use of multiple EMRs or changing EMRs in the middle of data collection (n = 2), and the challenge of case abstraction when urine culture results were delayed by 4–5 days after presentation (n = 1).

### Case reviews

Fifty-eight patient cases were reviewed by two physician reviewers (case characteristics shown in Table 3). By consensus clinical opinion (gold standard), 57% (33/58) were classified as ASB. By consensus ID-UTI assessment, 28% (16/58) of cases were ASB.

**Table 3.** Clinician demographics for each clinical case

	Patient cases n = 57* (%)
**Encounter type**	
Emergency department visit only	37 (65)
Admitted to hospital from emergency department or inpatient when urine culture obtained	18 (32)
Transferred to another hospital	2 (3)
**Cases in which ordering and treating clinician were the same person**	54 (95)
**Ordering clinician**^a^ **specialty**	
Family medicine	17 (30)
Emergency medicine	40 (70)
**Ordering clinician**^a^ **degree**	
Doctor of Medicine or Doctor of Osteopathic Medicine	49 (86)
Physician Assistant or Nurse Practitioner	8 (14)
**Treating clinician**^b^ **specialty**	
Family medicine	17 (30)
Emergency medicine	38 (67)
Internal medicine/hospitalist	2 (3)
**Treating clinician**^b^ **degree**	
Doctor of Medicine or Doctor of Osteopathic Medicine	49 (86)
Physician Assistant or Nurse Practitioner	8 (14)

*Clinician information was missing for one encounter

a Ordering clinician refers to the clinician that ordered the urinalysis and/or urine culture

b Treating clinician refers to the clinician who prescribed antibiotics

All cases classified as ASB by the ID-UTI assessment were classified as ASB by clinical opinion. In contrast, 17 cases classified as UTI by the ID-UTI assessment were classified as ASB by clinical opinion. These discrepancies occurred because of signs/symptoms that, upon review, were attributable to another cause. Urinary catheter malfunction causing obstruction was the most common reason for misclassification; remaining reasons are shown in Table 4. The most common diagnoses for cases determined to be ASB included urinary retention, intra-abdominal diagnoses, bilateral back pain, and fractures (Supplemental Table 1).

**Table 4.** Reasons that consensus ID-UTI classification differed from consensus clinical opinion, n = 17 (each line represents one patient case unless otherwise stated)

**Sign or symptom that qualified case as UTI by ID-UTI definition**	**Reason sign or symptom not considered UTI by physician reviewers**
Hematuria	Hematuria was isolated and most likely related to catheter trauma
Hematuria	Symptom was most likely attributable to vaginal bleeding
Hematuria and suprapubic tenderness	Symptoms most likely from blocked urinary catheter
Suprapubic pain (n = 2)	Symptom most likely attributable to clogged urinary catheter with urinary retention
Suprapubic pain and dysuria	Symptoms were completely resolved by urinary catheter placement so most likely from urinary retention
Dysuria	Patient had multiple non-urinary tract-specific symptoms (diffuse abdominal pain, vomiting) and recent surgery that suggested another cause
Dysuria	Symptom more likely due to yeast infection
Dysuria	Pain and urine passing around urinary catheter was more likely from malfunctioning urinary catheter
Flank pain	Flank pain was bilateral with absence of other urinary tract-specific symptoms
Flank pain	Initial complaint was abdominal pain, followed later in the course by bilateral flank pain without other urinary tract symptoms
Flank pain	Flank pain was bilateral and not accompanied by other urinary symptoms or imaging consistent with pyelonephritis
Fever (n = 2)	Patient had no urinary tract-specific symptoms at the time of presentation but did have another potential source (e.g. respiratory symptoms)
Fever and vital sign abnormalities	No urinary tract-specific symptoms and had diarrhea with abdominal pain, suggesting another cause
Altered mental status with vital sign abnormalities	Signs/symptoms most likely attributable to cardiac ischemia
Altered mental status and leukocytosis	Patient had altered mental status with mildly elevated white blood cell count, but many other potential reasons for this including opioid use (which may explain altered mental status)

UTI, Urinary tract infection; ID-UTI, “Inappropriate diagnosis of UTI”

### Validity

**Figure 1.**
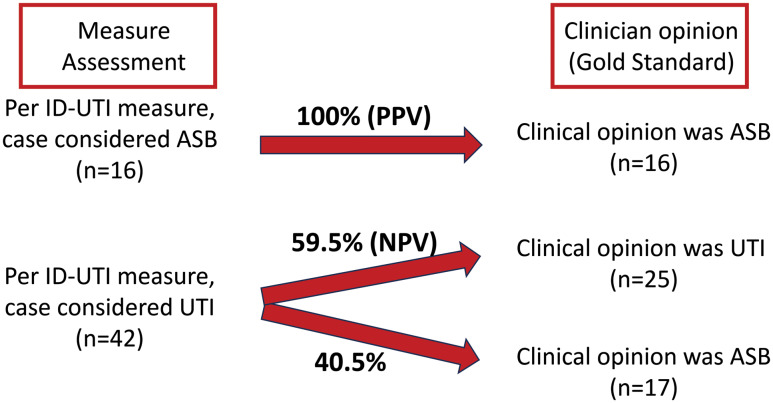
Positive predictive value (PPV) and negative predictive value (NPV) of the ID-UTI Measure vs Dual Physician Review. PPV is defined by the number of cases that were ASB by clinical opinion out of total cases that met ID-UTI definition of ASB; NPV is defined by the number of cases that were UTI by clinical opinion out of total cases that met ID-UTI definition of UTI. Abbreviations: UTI, urinary tract infection; ASB, asymptomatic bacteriuria; PPV, positive predictive value; NPV, negative predictive value.

Based on dual clinician review, the ID-UTI assessment had perfect specificity (100%, 95% CI 86.2–100; Figure 1)—ie if dual-physician review identified a case as UTI, it met the ID-UTI definition of UTI in 100% of cases. Similarly, if the ID-UTI assessment identified the case as ASB, it was ASB on physician review (PPV 100%, 95% CI 79.4–100). In contrast, sensitivity was poor (48.5%, 95% CI 31.4–65.6) indicating that the ID-UTI assessment failed to identify many cases of ASB; similarly, only 59.5% of cases called UTI were actually UTI (negative predictive value 59.5%, 95% CI 44.7–74.3).

Nine sites (90%) completed the pre-survey assessing face validity. All respondents reported that reducing antibiotic treatment for ASB was “very important” and 89% (8/9) of respondents “agreed” or “strongly agreed” that treatment of ASB was a relevant issue for their facility.

### Reliability

Clinician reviewers agreed with each other’s ID-UTI assessment 93.1% of the time (Cohen’s Kappa 0.82, strong agreement). There was also high reliability/agreement (93.1%, Cohen’s kappa of 0.83) between their ID-UTI assessment and the automated REDCap ID-UTI assessment.

## Discussion

As part of a year-long collaboration with 10 CAHs, we assessed the performance of a standard measure to assess ASB vs UTI in CAHs. Our data confirm that the ID-UTI measure could help identify inappropriate diagnosis and treatment of ASB and, by adding ED discharges, was feasible for some CAHs.

Similar to its performance in non-CAHs, when the ID-UTI measure identified a case as ASB, the patient was highly likely to have ASB based on physician review. In contrast, a case identified by the ID-UTI definition as UTI was frequently ASB (poor sensitivity). The reason for this discrepancy was that physician review was often required to ensure that potential UTI symptoms were in fact from a non-UTI cause (eg hematuria or suprapubic pain from catheter obstruction). Enhancing sensitivity (without worsening specificity) would require physician review for all cases—an infeasible strategy. Notably, the ID-UTI measure was designed as a pay-for-performance measure and thus designed to prioritize specificity over sensitivity (ie to accurately identify ASB)—it is critical in such measures that clinicians/hospitals trust that any “fallout” ASB case is highly accurate.^20^ Thus, the ID-UTI measure represents only the “tip of the iceberg” for ASB improvement. If an antibiotic stewardship program wished to increase measure sensitivity (ie identify all potential cases of ASB), they could consider removing some elements of the ID-UTI definition where clinical judgment is required (eg hematuria, urinary retention), but more review would be required to evaluate loss of specificity and ensure accuracy.

Our review of patient cases highlighted several unique findings related to ASB in CAHs. First, most patients were evaluated only in the ED without hospital admission; most clinicians empirically treated patients based on the results of their urinalysis rather than their urine culture. Second, almost all clinicians who prescribed antibiotics were the same ones who ordered urinary testing. Finally, urinary obstruction (commonly due to catheter malfunction) was the most common reason for treatment of ASB. Similar to larger hospitals, abdominal diagnoses, isolated altered mental status, and back pain were commonly misclassified by clinical providers as UTIs.^23^

Our findings have implications for national antimicrobial use measurement. Starting in 2024, CMS will require hospitals participating in the CMS Promoting Operability program, including CAHs, to submit antibiotic use (AU) and antibiotic resistance data to CDC’s National Healthcare Safety Network. Beyond requiring substantial information technology resources to collect and submit, interpretation of these metrics is challenging in CAHs for several reasons: small number of patients requiring antibiotics may cause significant variation in month-to-month reported days of therapy, patient populations may significantly differ between large hospital systems and CAHs, and a significant portion of antibiotic use may happen in the ED and therefore not be captured in AU data.^12^ Since the ID-UTI measure relies on case review rather than exclusively electronically-extractable data and can be used either in the ED or hospitalized patients, it may be more robust, particularly in CAHs where patient volume is low or EMRs are nonstandard and information technology resources are limited. In such cases, manual review may be preferable to electronic measurement. In a separately published manuscript, we found that using this ID-UTI measure to provide hospital-level feedback can reduce ASB treatment in CAHs (Ciarkowski et al, in press).

Use of the ID-UTI measure on a large scale would rely on the ability of hospitals, including CAHs, to perform regular case abstractions. The feasibility of case abstraction and adjudication in our study was mixed. Only 10 of 14 originally recruited hospitals were able to collect and submit data and a minority of sites were able to meet the requested number of case submissions to achieve high reliability. However, 70% of sites submitted sufficient cases for acceptable reliability, and all 10 sites achieved moderate reliability. Furthermore, our data have highlighted data elements that could be excluded from future case abstractions. A variety of reasons were responsible for inability to participate, which relate to challenges of small workforces with multiple responsibilities and limited resources, and inefficient or non-existent electronic tools to identify cases. If the ID-UTI measure was implemented as a pay-for-performance measure or for benchmarking, adequate resources and funding would be required for many CAHs to participate. However, most sites reported that this measure was as easy or easier than other quality improvement measures. Given the challenges of electronic data, chart review remains the primary option for QI assessment in CAHs.

Our study has limitations. Cases were adjudicated retrospectively by both case abstractors and physician reviewers based on available documentation. Second, the ID-UTI measure is designed to prioritize specificity and should not be used in situations where sensitivity is required (for example, use in real time by clinicians to determine UTI vs ASB). Third, sampling bias due to use of convenience sampling may have affected the submitted cases and we do not know the total number of urine cultures at each site. Fourth, the reliability thresholds, which determined the number of cases submitted by each hospital, were calculated based on estimates from non-CAHs and may not directly apply to CAHs. Fifth, we were underpowered to detect differences in validity or reliability in individual CAHs. Lastly, feasibility within this group of CAHs may not be generalizable to other CAHs, as the participating sites were highly motivated, and even within our initial group of CAHs, 4 were unable to continue participation. Face validity was also likely overstated as the CAH site champions who volunteered to participate may be more enthusiastic than non-volunteers. Study strengths include a multicenter design that drew from a large geographic area of CAHs, use of a previously validated definition of UTI vs ASB, and dual-physician case review to assess validity.

In summary, the ID-UTI measure was reliable and had high specificity but low sensitivity for identification of ASB when examined in 10 CAHs. Use of the ID-UTI measure for hospital peer comparison could help improve equity and antibiotic stewardship efforts in CAHs.

## Supporting information

Imlay et al. supplementary material Imlay et al. supplementary material

## Supplementary material

The supplementary material for this article can be found at https://doi.org/10.1017/ice.2024.206.
