# Supplementary material for: Validation and generalizability of an asymptomatic bacteriuria metric in critical access hospitals
Source: Infect Control Hosp Epidemiol. 2024 Dec 16;46(2):150–5. doi: 10.1017/ice.2024.206 (PMC11790320; doi:10.1017/ice.2024.206)
Supplement: Imlay et al. supplementary material [file S0899823X2400206Xsup001.pdf]

Supplement

Validation and Generalizability of an Asymptomatic Bacteriuria Metric in Critical Access Hospitals

Supplemental Table 1. Presenting symptoms/diagnoses among patients not clinically determined to have a urinary tract infection

Presenting symptom/diagnosis	Cases (n=33)
Urinary retention[a]	7
Abdominal diagnosis[b]	4
Back pain or bilateral flank pain as isolated symptom	3
Fractures[c]	3
Altered mental status as isolated symptom[d]	2
Fever as isolated symptom	2
Stroke	2
Poor appetite or failure to thrive as isolated symptom	2
Heart failure	1
Dizziness as isolated symptom	1
Vital sign changes	1
Myocardial infarction	1
Nausea/vomiting after recent surgery	1
Atrial fibrillation	1
Suspected yeast infection	1
Parainfluenza	1

[a]Urinary retention included 4 due to urinary catheter obstructions and 1 post-surgical case
[b]Abdominal diagnoses included gastroenteritis, diverticulitis, post-surgical complication, and stercoral colitis
[c]1 pelvic, 1 humeral, 1 spinal
[d]In one of the cases, altered mentation resolved by time of ED visit

CSiM IQIC_Utah Abstraction Form UTI

Please complete the survey below.

Thank you!

Hospital

- ○ Other
- ○
- ○
- ○
- ○
- ○
- ○
- ○
- ○
- ○
- ○
- ○
- ○
- ○
- ○

Other Hospital

____

Time abstractor begins case review:

____

Case Code: (Hospital-generated code (5 digits) in lieu of MRN - hospital retains link between case codes and MRNs)

____
(e.g., 00001, 00002, etc.)

Inclusion Criteria

	Yes	No
Age >/= 18 years	○	○
Positive urine culture (local lab definition; organisms listed in data dictionary)	○	○
Admitted to a non-ICU medicine service	○	○
Received any antibiotic intended to treat UTI during the symptom collection window (day -1, 0, 1, 2, where day 0 = day first positive urine culture was collected)	○	○
Immunocompetent or only mild immune compromise (Exclude if: hx of transplant; HIV and CD4< 200; neutropenia, ANC< 0.5; see data dictionary for details)	○	○

Treated only for UTI or infection secondary to UTI (e.g., sepsis, bacteremia, C Diff) during hospitalization	○	○
Have normal urinary anatomy (do not include patients with urinary stent, nephrostomy, altered urinary tract anatomy, or urologic surgery during hospitalization)	○	○

This case does not meet inclusion criteria. Please scroll to the bottom of this form and click 'submit' and move on to the next case review by clicking on the link (https://redcap.link/Case_Abstraction_Form).

Exclusion Criteria

	Yes	No
Patient left against medical advice (AMA) or refused care	○	○
Admitted on hospice	○	○
Women who are pregnant	○	○
Spinal cord injury	○	○
Had a UTI Complication (include any patient prescribed > 14 days of antibiotics)	○	○

This case is excluded. Please move on to the next case review. Please scroll to the bottom of this form and click 'submit' and move on to the next case review by clicking on the link (https://redcap.link/Case_Abstraction_Form).

Was the patient breastfeeding?

- ○ Yes
- ○ No
- ○ N/A
- ○ Unknown

Hospitalization

Location at the time of culture?

- ☐ Ambulatory care clinic
- ☐ ED, then discharged
- ☐ ED, then admitted
- ☐ Inpatient
- ☐ Rehab or long-term care facility
- ☐ urgent or quick care facility
- ☐ Other

What "Other" setting was the patient cultured in?

____

Was the patient sent to the ED or inpatient?

- ○ Yes
- ○ No

Was the culture reflexed from a urinalysis (UA)?

- ○ Yes
- ○ No
- ○ Unknown

NOTE: If possible, all case abstractions should be for patients treated in the hospital/acute care setting or the ED. If your hospital does not have enough of these cases and you have discussed this with the study team, then please continue with case abstraction. Otherwise, please select the next patient who was treated in the hospital/acute care setting or the ED.

Date of hospital encounter:
Date when the patient was first seen (e.g., in ED, clinic, etc) and may be before the date of hospitalization ______________________

Date of admission to hospital: ______________________

Date first positive urine culture was collected:
This is defined as Day 0 ______________________
(Date culture was collected, NOT the date result was reported)

Date of discharge from hospital: ______________________

Demographics

Age (years): ______________________

Sex
- ◯ Male
- ◯ Female
- ◯ Other or not specified

Race
- ◯ White
- ◯ Black
- ◯ Asian
- ◯ Pacific Islander
- ◯ American Indian or Alaska Native
- ◯ Multiracial
- ◯ Other
- ◯ Unknown or not specified

Ethnicity
- ◯ Hispanic/Latinx
- ◯ Not Hispanic/Latinx
- ◯ Unknown or not specified

Co-Morbidities (on Day 1 or 2 of hospitalization)

	Yes	No
Diabetes	◯	◯
End Stage Renal Disease (ESRD) - on dialysis	◯	◯
Dementia	◯	◯

Admitted from skilled nursing facility (SNF) or long-term assisted care facility (LTAC)	◯	◯
Immune suppression not severe enough to be excluded (e.g., HIV with CD4 > 200; taking low-dose steroids, < 20 mg prednisone/day; taking < 2 immunosuppressant agents)	◯	◯

Urologic Co-Morbidities

	Yes	No
Urologic procedure in 30 days prior to admission	◯	◯
Chronic urinary catheter use (i.e., foley)	◯	◯
Chronic intermittent straight catheterization	◯	◯
History of prostate cancer within the past year	◯	◯
Neurogenic bladder or urinary retention	◯	◯

SIRS criteria and organ dysfunction (check all that apply): Information from the period of -1 day to +2 days relative to urine culture (where Day 0 is day first positive urine culture was collected).

	Day -1	Day 0, [first_pos_uc]	Day 1	Day 2
No data are available for this day	☐	☐	☐	☐
Temp > 38.0 C	☐	☐	☐	☐
Temp < 36.0 C	☐	☐	☐	☐
HR > 90 bpm	☐	☐	☐	☐
RR > 20 breaths/minute	☐	☐	☐	☐
WBC > 10 K/uL	☐	☐	☐	☐
WBC < 4 K/uL	☐	☐	☐	☐
Hypotension (SBP < 90 mm Hg)	☐	☐	☐	☐
New organ dysfunction: Cr >2, Bilirubin >2, platelet < 100,000, INR >1.5, lactate > 2, OR SBP < 90 (see data dictionary for details)	☐	☐	☐	☐
None of the above (all negative)	☐	☐	☐	☐

Urinary Catheter
Information from the period of -1 day to +2 days relative to urine culture (where Day 0 is day first positive urine culture was collected).

	Day -1	Day 0, [first_pos_uc]	Day 1	Day 2
No data are available for this day	☐	☐	☐	☐
Urinary catheter in place	☐	☐	☐	☐
Intermittent straight catheter used	☐	☐	☐	☐
None of the above (both negative)	☐	☐	☐	☐

Signs and symptoms related to UTI:
Information from the period of -1 day to +2 days relative to urine culture (where Day 0 is day first positive urine culture was collected).

	Day -1	Day 0, [first_pos_uc]	Day 1	Day 2
No data are available for this day	☐	☐	☐	☐
Urgency	☐	☐	☐	☐
Rigors	☐	☐	☐	☐
Frequency	☐	☐	☐	☐
Dysuria	☐	☐	☐	☐
Suprapubic pain or tenderness	☐	☐	☐	☐
Acute hematuria	☐	☐	☐	☐
Costovertebral or flank pain or tenderness	☐	☐	☐	☐
Documentation of pyelonephritis	☐	☐	☐	☐
Fever (>38 C)	☐	☐	☐	☐
New onset mental status changes WITH systemic signs of infection (leukocytosis [WBC > 10uL] OR hypotension [SBP< 90 mmHg] OR 2 or more SIRS criteria)	☐	☐	☐	☐
New onset mental status changes WITHOUT systemic signs of infection (leukocytosis [WBC > 10 uL] OR hypotension [SBP< 90 mmHg] OR 2 or more SIRS criteria)	☐	☐	☐	☐
None of the above (all negative)	☐	☐	☐	☐

Micro Data

What bacteria is/are in the urine culture? (Select all that apply)	☐ Citrobacter species<br>☐ Coagulase-negative Staphylococcus spp.<br>☐ Enterobacter species<br>☐ Enterococcus species<br>☐ Escherichia coli<br>☐ Klebsiella species<br>☐ Staphylococcus aureus (MSSA or MRSA)<br>☐ Proteus mirabilis<br>☐ Pseudomonas aeruginosa<br>☐ Mixed flora<br>☐ Other
Other (primary urine culture organism)	__________________________________
Urinalysis:<br>Urinalysis that reflexed to culture or was taken just prior to primary urine culture	☐ Not performed<br>☐ Postive leukocyte esterase<br>☐ Positive nitrites<br>☐ Positive squamous<br>☐ Positive bacteria<br>☐ WBC marked positive in EMR but WBC < 10<br>☐ WBC > 10
Were any blood cultures obtained?	◯ Yes<br>◯ No
Blood culture results (mark all that apply):	☐ Positive; matched urine<br>☐ Positive; did not match urine - likely pathogen<br>☐ Positive; did not match urine - likely contaminant<br>☐ Negative
Blood culture organism	__________________________________<br>(only enter if different from urine culture)

Antibiotic Information

On what date did the patient first receive an antibiotic during the hospital encounter? (i.e., first day of antibiotics)	__________________________________
Did the patient receive IV antibiotics while inpatient?<br>(If patient received IM antibiotics while inpatient, please check 'yes')	◯ Yes<br>◯ No

Which IV antibiotics received during hospitalization? (Select all that apply)

- ☐ Ampicillin-sulbactam
- ☐ Cefazolin
- ☐ Cefepime
- ☐ Ceftriaxone
- ☐ Ciprofloxacin
- ☐ Ertapenem
- ☐ Levofloxacin
- ☐ Meropenem
- ☐ Metronidazole
- ☐ Piperacillin-tazobactam
- ☐ Trimethoprim-sulfamethoxazole
- ☐ Vancomycin
- ☐ Other

Which "other" IV antibiotics received during hospitalization? (Select all that apply)

- ☐ Amikacin
- ☐ Ampicillin
- ☐ Azithromycin
- ☐ Aztreonam
- ☐ Cefotaxime
- ☐ Cefotetan
- ☐ Cefoxitin
- ☐ Ceftaroline
- ☐ Ceftazidime
- ☐ Ceftazidime/avibactam
- ☐ Ceftolozone/tazobactam
- ☐ Cefuroxime
- ☐ Clindamycin
- ☐ Colistin
- ☐ Dalbavancin
- ☐ Daptomycin
- ☐ Doripenem
- ☐ Doxycycline
- ☐ Eravacycline
- ☐ Gentamicin
- ☐ Imipenem/cilastatin
- ☐ Linezolid
- ☐ Meropenem/vaborbactam
- ☐ Moxifloxacin
- ☐ Nafcillin
- ☐ Oritavancin
- ☐ Oxacillin
- ☐ Penicillin
- ☐ Piperacillin
- ☐ Televancin
- ☐ Tetracycline
- ☐ Tigecycline
- ☐ Tobramycin
- ☐ Other

Please type in which "other" IV antibiotic received during hospitalization.

____

Did the patient receive oral antibiotic therapy while inpatient?

- ◯ Yes
- ◯ No

Which oral antibiotics received during hospitalization? (Select all that apply)	☐ Amoxicillin<br>☐ Amoxicillin-clavulanate<br>☐ Cefdinir<br>☐ Cefixime<br>☐ Cefpodoxime<br>☐ Cefuroxime<br>☐ Cephalexin<br>☐ Ciprofloxacin<br>☐ Doxycycline<br>☐ Fosfomycin<br>☐ Levofloxacin<br>☐ Nitrofurantoin<br>☐ Trimethoprim-Sulfamethoxazole<br>☐ Other<br>( )
Which "other" oral antibiotics received during hospitalization? (Select all that apply)	☐ Azithromycin<br>☐ Clarithromycin<br>☐ Delafloxacin<br>☐ Dicloxacillin<br>☐ Linezolid<br>☐ Metronidazole<br>☐ Minocycline<br>☐ Other
Please type in "other" oral antibiotic received during hospitalization	__________________________________
Did the patient receive an IV antibiotic at discharge?	○ Yes<br>○ No
Which IV antimicrobials received at discharge? (Select all that apply)	☐ Ampicillin-sulbactam<br>☐ Cefazolin<br>☐ Cefepime<br>☐ Ceftriaxone<br>☐ Ciprofloxacin<br>☐ Ertapenem<br>☐ Levofloxacin<br>☐ Meropenem<br>☐ Metronidazole<br>☐ Piperacillin-tazobactam<br>☐ Trimethoprim-sulfamethoxazole<br>☐ Vancomycin<br>☐ Other

Which "other" IV antimicrobials received at discharge? (Select all that apply)	☐ Amikacin<br>☐ Ampicillin<br>☐ Azithromycin<br>☐ Aztreonam<br>☐ Cefotaxime<br>☐ Cefotetan<br>☐ Cefoxitin<br>☐ Ceftaroline<br>☐ Ceftazidime<br>☐ Ceftazidime/avibactam<br>☐ Ceftolozone/tazobactam<br>☐ Cefuroxime<br>☐ Clindamycin<br>☐ Colistin<br>☐ Dalbavancin<br>☐ Daptomycin<br>☐ Doripenem<br>☐ Doxycycline<br>☐ Eravacycline<br>☐ Gentamicin<br>☐ Imipenem/cilastatin<br>☐ Linezolid<br>☐ Meropenem/vaborbactam<br>☐ Moxifloxacin<br>☐ Nafcillin<br>☐ Oritavancin<br>☐ Oxacillin<br>☐ Penicillin<br>☐ Piperacillin<br>☐ Televancin<br>☐ Tetracycline<br>☐ Tigecycline<br>☐ Tobramycin<br>☐ Other
Please type in "other" IV antibiotic received at hospital discharge	__________________________________
Did the patient receive oral antibiotic therapy at discharge?	◯ Yes<br>◯ No
Which oral antibiotics received at hospital discharge? (Select all that apply)	☐ Amoxicillin<br>☐ Amoxicillin-clavulanate<br>☐ Cefdinir<br>☐ Cefixime<br>☐ Cefpodoxime<br>☐ Cefuroxime<br>☐ Cephalexin<br>☐ Ciprofloxacin<br>☐ Doxycycline<br>☐ Fosfomycin<br>☐ Levofloxacin<br>☐ Nitrofurantoin<br>☐ Trimethoprim-Sulfamethoxazole<br>☐ Other

Which "other" oral antibiotics received at hospital discharge? (Select all that apply)	☐ Azithromycin<br>☐ Clarithromycin<br>☐ Delafloxacin<br>☐ Dicloxacillin<br>☐ Linezolid<br>☐ Metronidazole<br>☐ Minocycline<br>☐ Other
Please type in "other" oral antibiotic received at hospital discharge	__________________
Completed antibiotic therapy while inpatient; no antibiotics prescribed at discharge?	◯ Yes<br>◯ No
Total inpatient IV antibiotic days	__________________<br>(calendar days)
Total inpatient oral antibiotic days	__________________<br>(calendar days)
Total inpatient antibiotic days (IV + oral)	__________________<br>(calendar days, including both IV and oral)
Total discharge antibiotic days	__________________<br>(calendar days)
Total treatment antibiotic duration (days)	__________________<br>(x.x days)
Time abstractor finishes case review:	__________________
Total abstraction time:	__________________
